# An *In Situ* Study of Precursor Decomposition via Refractive Index Sensing in p-Type Transparent Copper Chromium Oxide

**DOI:** 10.1021/acs.chemmater.1c03910

**Published:** 2022-03-18

**Authors:** Ainur Zhussupbekova, Kuanysh Zhussupbekov, Ruggero Verre, David Caffrey, Kyle Shiel, Igor V. Shvets, Karsten Fleischer

**Affiliations:** † School of Physics and Centre for Research on Adaptive Nanostructures and Nanodevices (CRANN), Trinity College Dublin, Dublin 2, Ireland; ‡ Department of Physics, Chalmers University of Technology, Gothenburg 412 96, Sweden; ¶ School of Physics, Dublin City University, Dublin 9, Ireland

## Abstract

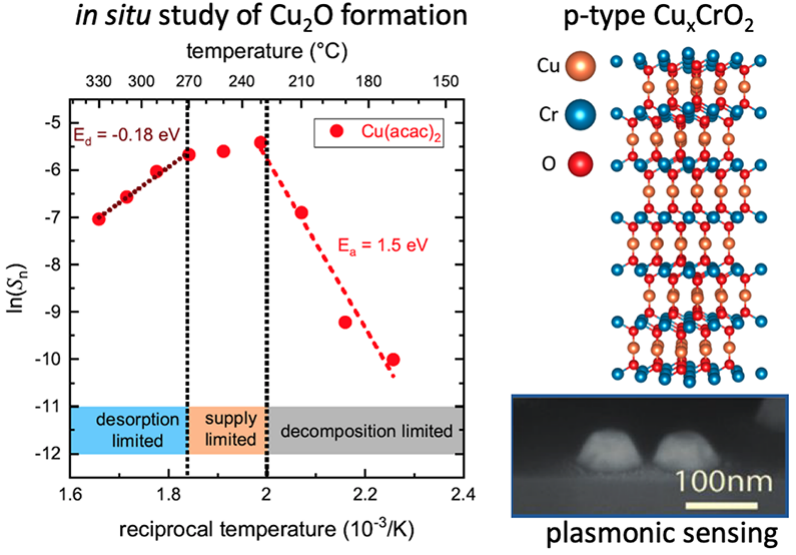

Oxide semiconductors are penetrating into a wide range of energy, environmental, and electronic applications, possessing a potential to outrun currently employed semiconductors. However, an insufficient development of p-type oxides is a major obstacle against complete oxide electronics. Quite often oxide deposition is performed by the spray pyrolysis method, inexpensive to implement and therefore accessible to a large number of laboratories. Although, the complex growth chemistry and a lack of *in situ* monitoring during the synthesis process can complicate the growth optimization of multicomponent oxides. Here we present a concept of plasmonic, optical sensing that has been applied to spray pyrolysis oxide film growth monitoring for the first time. The proposed method utilizes a polarization based refractive index sensing platform using Au nanodimers as transducing elements. As a proof of concept, the changes in the refractive index of the grown film were extracted from individual Cu(acac)_2_ and Cr(acac)_3_ precursors in real time to reveal their thermal decomposition processes. Obtained activation energies give insight into the physical origin of the narrow temperature window for the synthesis of high performing p-type transparent conducting copper chromium oxide Cu_*x*_CrO_2_. The versatility of the proposed method makes it effective in the growth rate monitoring of various oxides, exploring new candidate materials and optimizing the synthesis conditions for acquisition of high performing oxides synthesized by a high throughput cost-effective method.

## Introduction

Transparent conducting oxides (TCOs) are materials possessing a rare combination of two contradicting properties of electrical conductivity and optical transparency in a single material. Exhibiting this remarkable coexistence of properties, TCOs are desired materials in a broad range of applications including solar cells, flat panel displays, touch screens, light emitting diodes, transparent electronics, memristors, and neuromorphic applications

The most common and better performing TCOs are n-type, while the development of high performing p-type TCOs remains an outstanding challenge due to low hole density, localized nature of the valence band derived of O 2p, and large hole effective masses.^1,2^ Despite current limitations, p-type TCOs have been already used to selectively extract and transport p-type charge carriers^3^ in thin film solar cells^4,5^ and organic light emitting diodes and have been also used as a UV-blocking layer to improve the photostability of dye sensitized and perovskite solar cells.^6^ These applications are a consequence of the wide band gap of p-type TCOs, their thermal stability, a high work function, and good hole conductivity.^7^ To best serve these technologies, p-type TCOs have to display an appropriate valence band structure, well aligned with the high work function of a contact and the valence band^8^ of the photoactive layer to effectively suppress diffusion between the electrode material and the photoactive layer.

A class of p-type TCO material with the potential to solve the issue of low performance is copper-based delafossite oxides with a general formula CuMO_2_ in which M corresponds to a trivalent cation such B, Al, Cr, Ga, Sn In, or Y. The delafossite structure is composed of each Cu atom linearly coordinated with two O atoms, forming an O–Cu–O dumbbell parallel to the *c*-axis. Every oxygen anion is also coordinated to M^3+^ cations oriented in a way that the M-centered octahedra form MO_2_ layers that are parallel to the *ab* plane.^1^ Usually Cu-based delafossites have higher conductivity values yet lower Hall mobility values.^9^ This higher conductivity is possibly justified by the hole concentrations produced by a higher density of native acceptor-like defects.^1^ One notable exception within the class of copper delafossites is CuCrO_2_,^10^ where the top of the valence band was found to be formed by the Cr 3d states rather than the Cu 3d state.^11−13^ This facilitates an insensitivity to the crystalline quality as the conduction is based on a hopping mechanism between CrO_2_ octahedra, allowing for nanocrystalline or even amorphous p-type TCOs grown at much lower temperatures than conventional copper delafossites.

Previously a very narrow processing window for spray pyrolysis grown, best performing p-type Cu_*x*_CrO_2_^14,15^ was reported. In order to analyze the origin of this narrow temperature window for Cu_*x*_CrO_2_ synthesis, a concept of plasmonic, optical sensing is applied to spray pyrolysis for the first time, allowing *in situ* oxide film growth monitoring by probing of the activation energies of the film formation process. The gradual changes of the effective medium between the Au nanoparticles from air to Cu_*x*_CrO_2_ during film formation were monitored using a polarization-based *in situ* optical sensing technique for a variety of growth temperatures. This methodology was tested using two widely employed^16^ Cu(acac)_2_ and Cr(acac)_3_ precursors, yet the versatility of the technique allows for it to be applied to real time growth monitoring of various oxides. To further explore the tunability of Cu_*x*_CrO_2_ films, the influence of temperature and oxygen partial pressure on the film composition and electrical performance was investigated.

## Methods

### Gold Nanoparticles Preparation

The Au dimers are made by hole–mask colloidal lithography.^17^ To investigate the spray pyrolysis process, substrate temperatures of up to 400 °C are required. To minimize any changes in the Au-dimer shape and size, during the actual measurements all Au-dimer substrates are pretreated by coating with a 2 nm thin Al_2_O_3_ film grown by atomic layer deposition (Oxford Instruments, 250 °C, 20 cycles of 20 ms pulse of TMA precursor, each followed by Ar purge and water pulse). Fuctionalized substrates were preheated to 400 °C prior to material deposition and optical sensing.

### Spray Pyrolysis Using Medical Nebulizers

p-Type Cu_*x*_CrO_2_ oxide films were deposited on standard microscope glass slides (Thermo Scientific 2.5 × 5 cm, 1 mm thick) by spray pyrolysis. In contrast with the commonly employed air-blast nozzle modified setup, we employ a medical grade nebulizer positioned in a small chamber with an optical window, used for *in situ* monitoring. The same pyrolytic decomposition of the dissolved precursors, sprayed by the stream of carrier gas toward the surface of a substrate, governs the deposition. In contrast to conventional spray pyrolysis, the different nebulization method of the solution allows the formation of high-quality, smooth films at lower substrate temperatures. In a conventional spray pyrolysis system, MAD-0331-B1B Ultrasonic Atomisers were employed; they tend to produce a broad range of droplet sizes ranging from ≈10–30 μm.^18^ The average droplet sizes produced by mist chemical vapour deposition (CVD) are found to be around 5.4 μm.^19^ The main difference is a reduction in the active cooling from the nebulizing gas, as much lower gas flow rates are required. In addition, the more homogeneous droplet size distribution and the reduced impact damage from larger droplets accelerated onto the film by the high pressure in air blast nebulizers further improve the film morphology.

The liquid precursor solution was prepared by dissolving Cu(acac)_2_ and Cr(acac)_3_ in methanol with a 70:30 Cr/Cu ratio, an overall total molarity of 0.03 M,^20^ and a gas flow of 4 L/m. The choice of precursors is based on characteristics such as volatility, thermal stability, and reactivity.^21,22^ The solution was nebulized by a Hudson RCI Micro Mist nebulizer creating a “mist”, which is transported toward the heated substrate (Watlow CER-1-01-00250 Ultramic ceramic heater). The temperature triggers the evaporation of the solvent while vaporized precursors remain in a gaseous state to form a desired material on the substrate surface.^23^ A range of processing temperatures were tested, and the optimum temperature for good conductive Cu_*x*_CrO_2_ was found to be 310 °C, approximately 40 °C lower than in our conventional air-blast nebulizer system using the same precursors.^14,15^

The precursor activation energy study was conducted in the same chamber, as it is equipped with a window for *in situ* optical sensing. Each precursor was studied separately in the temperature range from 230 to 390 °C. The film formation was monitored by observing changes in the localized surface plasmon resonance (LSPR) of anisotropic gold dimers.^24^

### X-ray Photoelectron Spectroscopy

The film composition was measured by X-ray photoelectron spectroscopy (XPS) in an Omicron MultiProbe XPS using a monochromized Al Kα source (XM 1000, *E*_*hν*_ = 1486.7 eV). Prior to XPS measurements films were sonicated in acetone and isopropanol for 10 min and then etched via an argon ion gun operated at a low voltage of 750 eV with a sputter current of ≈6 μA and chamber pressure of 1.5 × 10^–3^ Pa to ensure removal of unreacted precursors. Representative XPS scans are shown in the Supporting Information (SI).

### Raman Spectroscopy

Raman spectra of Cu_*x*_CrO_2_ samples were recorded using a JY Horiba LabRAM800 confocal micro-Raman setup with motorized positioning stages. The excitation wavelength was 488 nm, power ∼ 10 mW, and the integration time was 5 min. To maximize the Raman signal from the thin films, the underlying substrate was numerically subtracted for each spectrum. Details of the subtraction method were previously reported.^25^

### Optical Characterization

Optical properties were measured via UV–visible spectrophotometry (UV–vis) on a PerkinElmer S650 instrument.

### Electrical Properties

The carrier activation energy is determined from the four point linear probe measurements using a Keithley 2400 source meter. Seebeck measurements were performed as previously described^13^ (see SI).

### Thickness Measurements and Modeling

The thickness of each film was determined by X-ray reflection (XRR) (Bruker D8-Discover) as well as by optical modeling of UV–vis transmission and reflectance spectra. By dividing the film thickness with the growth time, the average growth rate for each film was determined in absolute terms of nm/min.

## Results and Discussion

### Methodology of *In Situ* Spray Pyrolysis Growth Monitoring

The synthesis of any ternary material is a complex process. The possibility of monitoring the properties of the films *in situ*, during the growth, enables the determination of vital information, such as the refractive index of the material and the growth mechanisms. For this reason, reflectance based optical techniques are frequently employed.^26−29^ However, in the spray pyrolysis the nebulized solution generates large scattering and droplet absorption along the light path within the chamber, rendering standard intensity-based reflectance measurements challenging.

**Figure 1:**
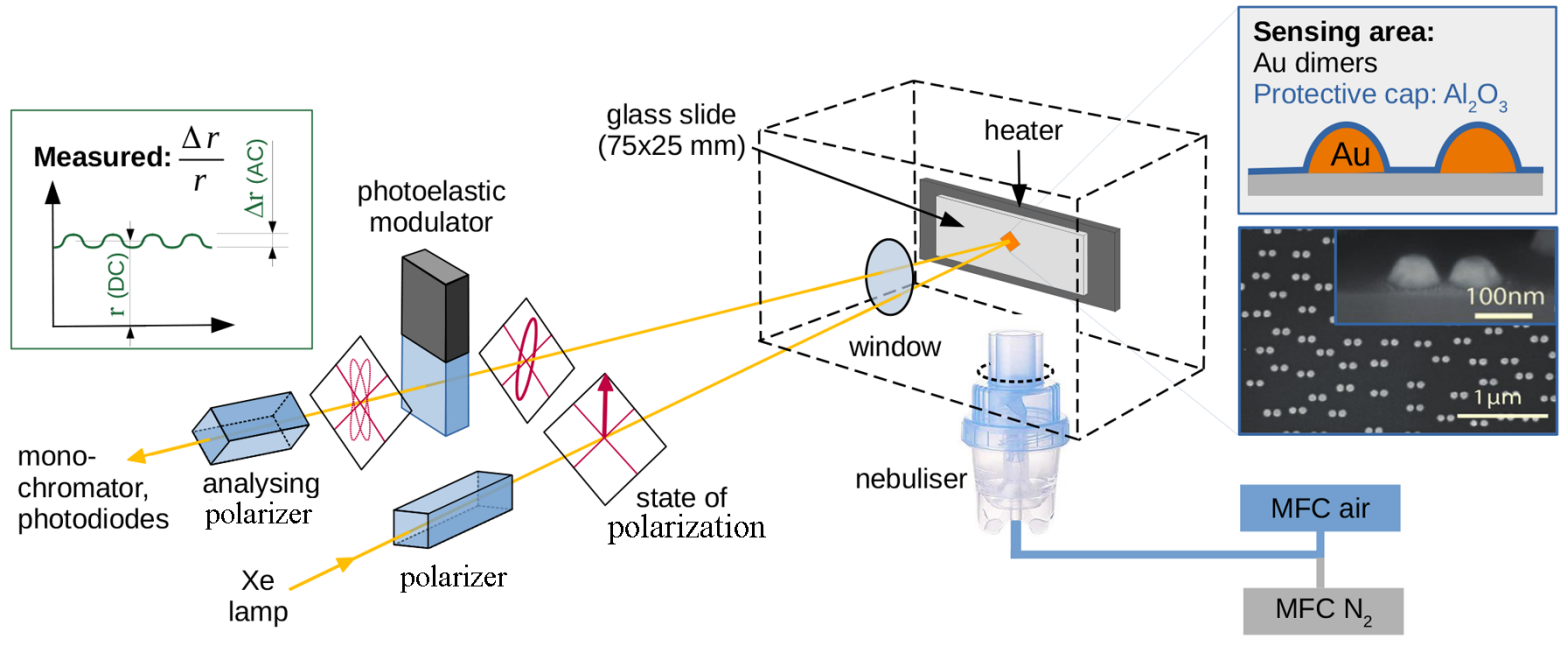
Schematic of the small spray pyrolysis chamber and the main optical components of the RAS setup. The inset shows the SEM image of Au dimers before Al_2_O_3_ capping.

In order to allow *in situ* measurements of the spray pyrolysis process, a sensing approach based on polarization based refractive index sensing was adapted.^24^ Previously, the method was used for optical biosensing that allowed the analyzation of molecular interactions at the nanoscale. However, the usage of plasmonic nanoparticles for oxide growth sensing has never been performed before. Therefore, a small deposition chamber was specifically designed with an optical window normal to the sample surface as schematically demonstrated in Figure 1. The sample was clamped on a vertically mounted heater with the nebulizer placed directly underneath, allowing the use of low cost medical nebulizers. The gas supply was controlled by mass flow controllers (MFCs) to allow for a controlled variation of the oxygen partial pressure, as well as reproducible gas flow rates into the nebulizer. The entire setup was mounted on an optical alignment stage allowing for adjustment of the reflected beam more precisely. The material was deposited on a glass slide with 10% surface area covered by plasmonic Au nanodimers, all aligned along the same direction. An electron micrograph of a typical surface is shown in the inset in Figure 1. The morphological anisotropy of the plasmonic nanostructure induces an optical anisotropy in the glass substrate, as the light is absorbed differently along and perpendicular to the dimer axis. When linearly polarized light is sent at a normal incidence of 45°, rotated with respect to the dimer axis, the optical anisotropy causes a rotation of the polarization axis of the reflected beam. If light is sent at a normal incidence, the rotation angle of light is proportional to the reflectance anisotropy of the surface using the small angle approximation sin θ = θ
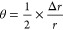
where *r* is the complex reflectance of the sample along the *i*th direction;  is also known as reflection anisotropy spectroscopy (RAS), and it is a self-normalized quantity that monitors the relative change in intensity between two orthogonal directions. Details and the specific use in refractive index sensing are found elsewhere.^24,30,31^

**Figure 2:**
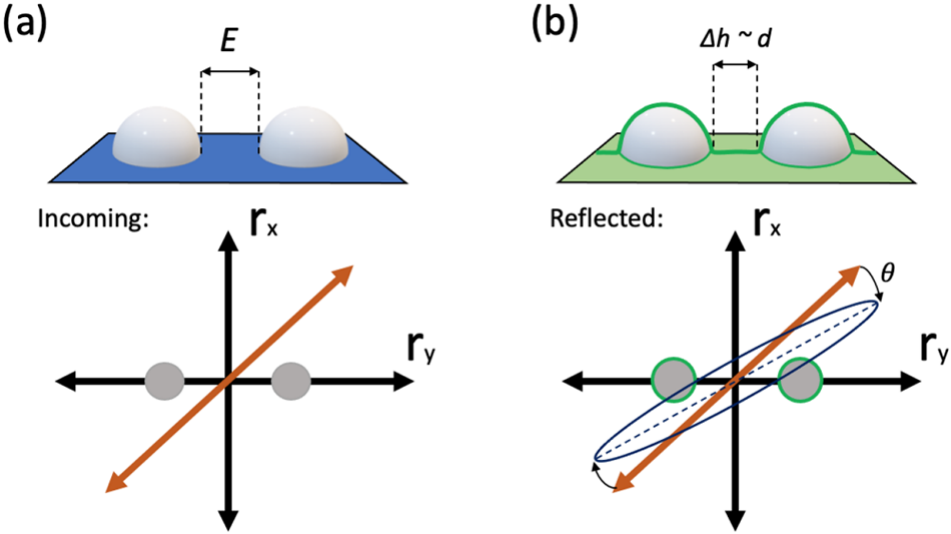
RAS signal acquisition from gold dimers (a) before deposition and (b) after deposition.

The strong RAS signal from Au dimers arises from a difference in polarizability along and perpendicular to dimers direction as is schematically demonstrated in Figure 2a. Therefore, the Au nanodimers act as local optical transducers retrieving the optical properties of the TCO growth *in situ*. In fact, not only do the Au dimers induce a rotation of the polarization angle of the polarized light, but they are extremely sensitive to their surrounding refractive index (*n*) and the thickness (*d*) of the deposited film as is shown in Figure 2b. The plasmonic nanostructure can be used to extract indirect information on the spray pyrolysis film characteristics. Increasing the refractive index between the nanoparticles in a subsequent growth run will, in a first order approximation, shift the plasmonic resonance toward the infrared due to an increase of the effective medium surrounding the Au nanoparticles.

To allow for a simple comparison between different spectra, the transient of RAS as a function of time is monitored in the region of largest sensitivity of the spectra, at 1.85 eV (see SI for details on the normalization procedure). Due to the self-normalizing nature of the RAS measurement, variations in total intensity caused by fluctuations and turbulence in the nebulizer mist do not effect the spectra. In principle, the setup also allows for measurements of the total reflectance, yet increased noise levels due to the mentioned fluctuations in the droplets around the sample, as well as condensation of unreacted precursor on the inside of the window, limits the use in the simple reflectance mode.

**Figure 3:**
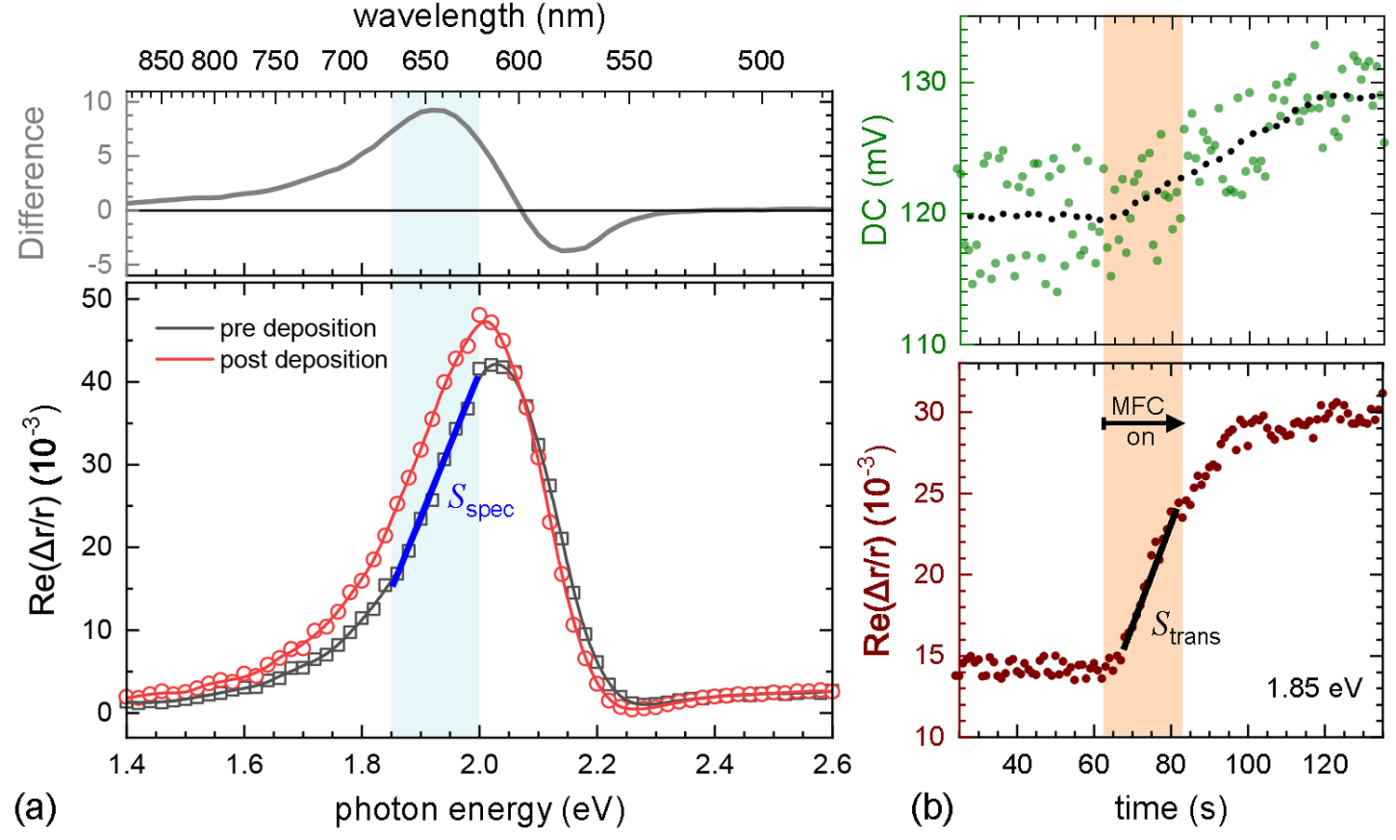
Example of a deposition cycle. (a) RAS spectra of the LSPR meta-surface at 230 °C before and after a deposition cycle using a 0.01 M solution of Cu(acac)_2_ in methanol. The top panel shows the difference in the two spectra. The shift in the spectral position of the plasmon resonance is evident. In the shaded regions there is a large difference and a nearly linear spectral dependence. Single wavelength, transient measurements during depositions are best taken there. (b) Transient measurement at 1.85 eV taken during growth. The shaded area indicates the time when the gas supply to the nebulizer was on. The top panel shows the DC level of the measurement proportional to the intensity of the reflected light.

Figure 3 shows a RAS spectrum of a stabilized localized surface plasmon resonance (LSPR) of an anisotropic gold dimer meta-surface acquired before and after a deposition step *S*_spec_ (a), as well as a transient *S*_trans_ (b) during the deposition. The rise in the RAS signal during the transient measurement shows an increase in material deposited over and between the gold dimers. Normalization of the latter with the spectral slope provides a renormalized value *S*_*n*_ = *S*_trans_/*S*_spec_ that is proportional to the product of the film growth rate and refractive index, measured for the temperature and precursor combination chosen for investigation. For ultrathin films, all optical methods are only sensitive to the product of the refractive index and thickness (*n* × *d*) due to the fundamental nature of light interaction at interfaces.^32,33^

For our analysis we assume that *n* does not significantly vary for the material formed at the different temperatures. While this is a simplification, and the refractive index of the material may easily vary by 10% within the first 2–10 nm,^34,35^ a change in thickness from 1 to 2 nm changed *d* by 100%. Hence the thickness change will dominate the response. Therefore, our methodology is beneficial in an optimization and fast screening of the synthesis of novel multicomponent oxides as well as in understanding the growth mechanism and chemistry of the spray pyrolysis process.

### Precursor Decomposition Screening for Copper Chromium Oxide

Thermal decomposition studies of acetylacetonate precursors have found that the Cu(acac)_2_ precursor starts to decompose at 200 °C while the corresponding Cr(acac)_3_ starts to decompose at 250 °C.^36^ More recent work has shown that the Cr(acac)_3_ precursor will have lost at least one (acac) group at 280 °C.^37^ However, the precursor decomposition is only the first step in the spray pyrolysis growth process. It then also involves subsequent adsorption of the precursor onto the surface, either in an unreacted form or in a partially decomposed form. The adsorption itself will depend on the surface termination and temperature. Indeed, it is already known that Cr(acac)_3_ adsorption can be self-limited, preventing a continuous growth at low temperatures. Due to these properties, Cr(acac)_3_ is used as an atomic layer deposition precursor, where this self-limitation is employed and Cr_2_O_3_ is formed with subsequent oxidation steps.^38^ In spray pyrolysis the presence of oxygen in the chamber, as well as OH groups and H_2_O from the further pyrolytic decomposition of the precursors and solvents, can lead to continuous reactions and film formation.

**Figure 4:**
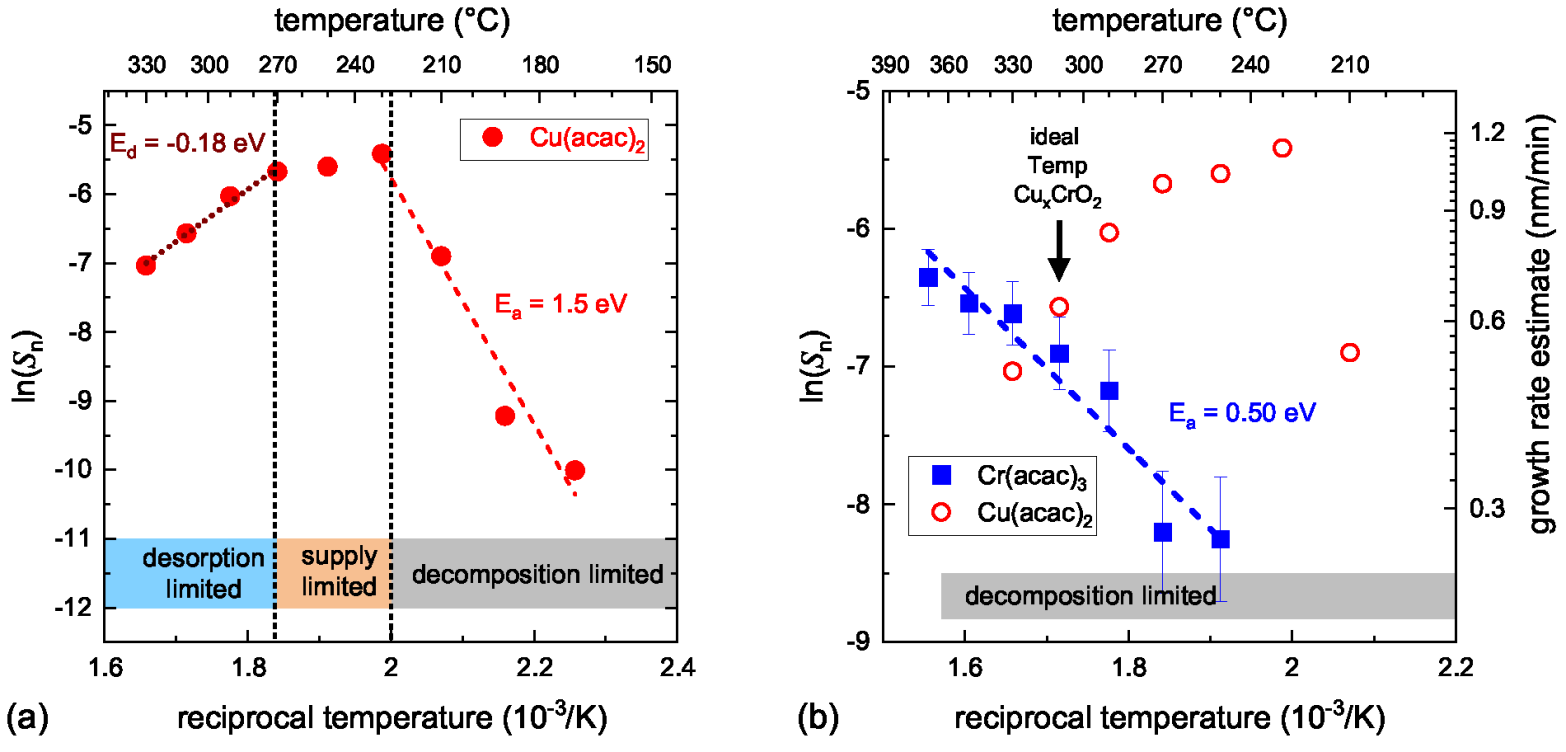
Arrhenius type plots of the normalized slope *S*_*n*_ for (a) the Cu(acac)_2_ precursor (0.01 M solution). (b) Comparison of the Cr(acac)_3_ precursor (0.015 M solution) measurement to those of Cu(acac)_2_. Both measurements were performed with 5% oxygen content in the nebulizing gas. The estimated growth rate scale in (b) is given as a rough guide and is based on an assumed linear relationship of the growth rate with molarity and *S*_*n*_.

By probing the buildup of the fully formed oxide layer around the plasmonic nanoparticles, it is possible to examine the thermodynamics of the entire spray pyrolysis process. As discussed above, one can determine a normalized slope *S*_*n*_ for each precursor and temperature. Plotting log(*S*_*n*_) in an Arrhenius type graph vs 1/*T* shows characteristic differences between the Cr and Cu precursors that are depicted in Figure 4.

As expected from the thermal decomposition studies, the Cu precursor is more reactive at lower temperatures than the Cr precursor. For the Cr precursor a linear slope is seen, giving an effective activation energy for the Cr_2_O_3_ film formation of 0.5 eV. The Cu precursor, in contrast, displays a more complex behavior. At low temperatures the onset of growth is observed, limited by the actual decomposition of the precursor with an activation energy of 1.5 eV. Once the precursor fully decomposes, the kinetically limited regime is reached, where the growth rate depends on the solution molarity and oxygen supply. Increasing the temperature further actually reduces the growth rate significantly. This is characterized by an activation energy for desorption of 0.18 eV. Either the copper suboxides thermally desorb, there is a depletion of the precursor in the gas phase, or the formation of acetic acid in the precursor decomposition at higher temperatures^36^ leads to an active etching of the formed Cu_2_O.^39^

The observed behavior for the decomposition and oxide film formation for the individual Cu and Cr precursors now directly explains the narrow growth window for Cu_*x*_CrO_2_. For temperatures lower than the optimum 310 °C, the Cr precursor does not sufficiently decompose and only Cu_2_O is formed independently of the actual Cu molarity in the solution. Increasing the temperature reduces the Cu incorporation dramatically either by Cu suboxide desorption or by depletion of the precursor in the gas phase (see Figure 4a), while also significantly increasing the Cr incorporation. In order to enlarge the temperature processing window for low cost spray pyrolysis of this specific material, one would need to find a Cr precursor that decomposes more readily at lower temperatures.

Our findings demonstrate that Cr_2_O_3_ is only formed substantially above 330 °C, yet the ideal growth temperature for the ternary Cu_*x*_CrO_2_ is 310 °C. The low temperature regime (<330 °C) is also likely to be dominated by the adsorption of unreacted Cr(acac)_3_ rather than actual Cr_2_O_3_ formation. We observe no film growth in this range when using higher molarity solutions, in line with self-limited adsorption^38^ Therefore, the presence of Cu suboxides must alter the Cr(acac)_3_ adsorption or on-surface decomposition. In mixed solutions, the *in situ* optical monitoring is of less use, as it does not distinguish compositions of the growing film. Therefore, to confirm these findings, thicker samples grown for a longer time of 10 min and higher molarity of 0.03 M at 310 °C were considered. The resulting film thicknesses were measured to determine the overall growth rate. Using solutions of 0.03 M Cu(acac)_2_, the growth rate of 1.5 ± 0.3 nm/min for binary Cu_2_O was obtained. The growth rate for a corresponding Cr_2_O_3_ film using 0.03 M Cr(acac)_3_ is found to be lower at 0.8 ± 0.2 nm/min. The ratio of 1.8 ± 0.4 between these growth rates is in agreement with the estimation from the *in situ* study where a ratio of 2 was expected based on the values of *S*_*n*_ at 310 °C. Films grown with a *mixture* of Cu(acac)_2_ and Cr(acac)_3_ (ratio 40/60), while keeping the total molarity at 0.03 M, have a significantly higher growth rate of 2.4 ± 0.4 nm/min. Clearly the presence of Cu suboxides on the surface dramatically increases the Cr incorporation at this temperature, and such an effect has been seen for other ternary oxide growth.^40,41^ Indeed the film is almost twice thicker than expected for a pure linear combination of the Cu_2_O and Cr_2_O_3_ growth rate.

### Tunability of Copper Chromium Oxide via Oxygen Partial Pressure

**Figure 5:**
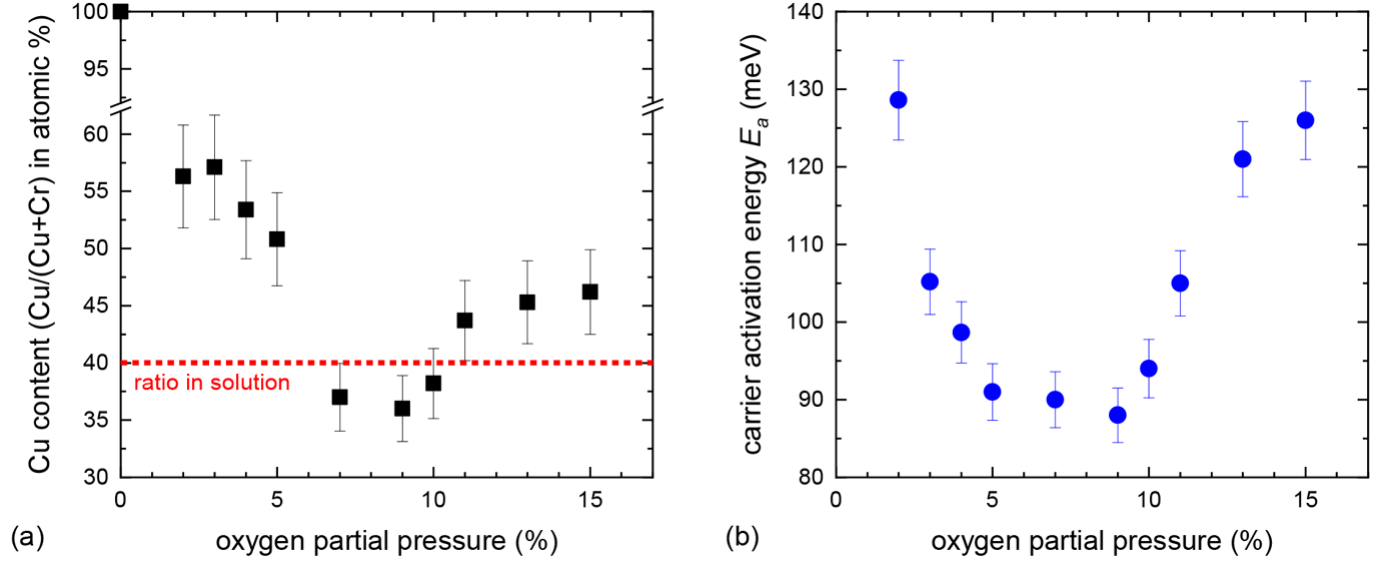
Film composition (a) and carrier activation energy (b) as a function of oxygen content in the nebulizing gas. Each sample was deposited until the full cup of 8 mL is emptied at a temperature of 310 °C.

To further explore the possibilities of Cu_*x*_CrO_2_ tuning, an in-depth analysis of the oxygen role in the film formation was conducted. Figure 5a shows the stoichiometry of bulk films grown from the same solution as a function of oxygen partial pressure. Growing in full nitrogen leads to the formation of an insulating Cu_2_O film, confirmed via XPS and Raman spectroscopy, while supplying only 2% of oxygen creates the condition for Cu_*x*_CrO_2_ formation. Instrumental limitations of the MFCs prevented investigations in the 0–2% range. A further increase in oxygen initially further increases Cr incorporation, and between 5% and 10% oxygen content in the nebulizing gas the film stoichiometry matches the solution stoichiometry. Increasing the oxygen content above 10% inhibits Cr incorporation again, and at the same time films become more resistive with a noticeable increase in carrier activation energy as seen from Figure 5b.

As temperature and solution stoichiometry remain unchanged, this suggests that the Cr precursor preferentially adsorbs on the Cu–O terminated surface in oxygen poor conditions. Insufficient oxygen supply prevents the adsorption of Cr precursor at 310 °C as seen in the sole formation of Cu_2_O in this case. At the same time, an excess of oxygen is detrimental to Cr adsorption as the films get thinner and more Cu rich again (see Figure 5a). This may suggest an overoxidized Cu–O layer possibly creating CuO instead of Cu_2_O. Indeed, studies of Cu(acac)_2_ decomposition in atmospheric CVD reactors have shown CuO formation when a carrier gas mixture of 10% O_2_/N_2_ has been used, although experiments were done at higher temperatures.^42^ Hence, these conditions are likely detrimental to Cu_*x*_CrO_2_ formation where Cu is in the same charge state as in Cu_2_O. At the same time one also needs to prevent the formation of oxygen vacancies and, as discussed above, oxygen is required for the incorporation of the Cr. Both effects combined explain the overall dependency of film stoichiometry and film conductivity on the oxygen content. The optimum oxygen content in the nebulizing gas is therefore highly dependent on the instrument, as in larger chambers more residual oxygen in the surrounding atmosphere can influence the growth.

**Figure 6:**
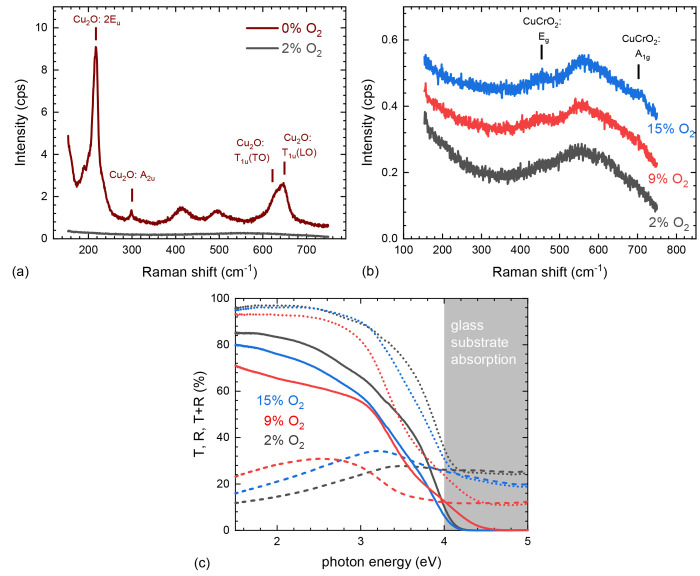
Raman spectra of films grown at varying oxygen content in the nebulizing gas: (a) full nitrogen and 2% oxygen and (b) 2%, 9%, and 15% oxygen. Labels indicate expected peak positions for highly crystalline materials. (c) *T* (solid lines), *R* (dashed lines), and *T* + *R* (dotted lines) of the same films. Changes are dominated by differences in overall film thickness, with 9% being the thickest.

The dramatic change in film composition upon the change of nebulizing gas from full nitrogen to a gas mixture that contains oxygen is most noticeable in the Raman spectra (shown in Figure 6a,b), where for the nitrogen case strong Cu_2_O Raman modes are observed, while films containing oxygen only demonstrate very weak and broad modes consistent with the nanocrystalline nature of the Cu_*x*_CrO_2_. The expected position for the *E*_*g*_ and *A*_1*g*_ Raman modes for crystalline Cu_*x*_CrO_2_ are 460 and 710 cm^–1^, respectively.^43^ The increase in oxygen does not change the position yet slightly sharpens the Raman modes, indicating a less defective material. This is consistent with the increased carrier activation energy and higher resistivity of the samples grown in higher oxygen content, as the Cu-vacancy defect structures are crucial for the conductivity in this material. The Cu_2_O Raman spectra of the nitrogen grown sample is dominated by a second-order line at 218 cm^–1^ and the TO and LO phonon modes of a typically Raman inactive mode (*T*_1*u*_). The latter was shown to be increased by point defects, specifically Cu vacancies.^44^

Figure 6c shows transmission (*T*) and reflection (*R*) of Cu_*x*_CrO_2_ films deposited at various oxygen partial pressures. While we see variation in transmission and reflection, they are largely originating from the changed film thickness rather than substantial changes in the optical properties of the material.

In conclusion, it was demonstrated for the first time that plasmonic sensing is a viable technique for *in situ* growth information acquisition in spray pyrolysis. In particular, refractive index sensing by RAS using plasmonic meta-surfaces to monitor the film growth was performed. Analysis of precursor decomposition and film formation for Cu and Cr precursors allowed the origin of the narrow synthesis window of Cu_*x*_CrO_2_ to be identified. Moreover, upon supplying both precursors simultaneously, a Cu induced uptake of Cr incorporation is observed. The produced Cu_*x*_CrO_2_ films display low roughness, unusual for inexpensive solution process deposition, confirming that medical grade nebulizers create gentle mist CVD-like growth. The alteration of the oxygen content can be effectively employed to further tune the composition and electrical performance of the films. These results expand the understanding of the synthesis mechanism and doping in ternary copper chromium delafossite. Beyond this specific material, the developed real time growth monitoring method will be applicable for many other spray pyrolysis processes and can quickly identify ideal growth conditions for various ternary oxides or assist in finding alternative precursors to further reduce growth temperatures to minimize the thermal budget of thin film synthesis.

## Supporting Information Available

Additional information on the *in situ* optical method employed, as well as measurements and properties of the Cu_*x*_CrO_2_ films such as X-ray reflection, details of the electrical characterization, and results (Seebeck, carrier activation energy) as well as representative XPS scans illustrating how the sample stoichiometry was determined (PDF)
