# Supplementary material for: An In Situ Study of Precursor Decomposition via Refractive Index Sensing in p-Type Transparent Copper Chromium Oxide
Source: Chem Mater. 2022 Mar 18;34(7):3020–7. doi: 10.1021/acs.chemmater.1c03910 (PMC9009087; doi:10.1021/acs.chemmater.1c03910)
Supplement: Supplementary file 1 — cm1c03910_si_002.pdf [file cm1c03910_si_002.pdf]

An in situ study of precursors decomposition via refractive index sensing in p-type Transparent Copper Chromium Oxide

Supporting Information

Ainur Zhussupbekova,*,† Kuanysh Zhussupbekov,† Ruggero Verre,‡ David Caffrey,† Kyle Shiel,¶ Igor V. Shvets,† and Karsten Fleischer¶

†*School of Physics and Centre for Research on Adaptive Nanostructures and Nanodevices (CRANN), Trinity College Dublin, Dublin 2, Ireland*
‡*Department of Physics, Chalmers University of Technology, Gothenburg, Sweden*
¶*School of Physics, Dublin City University, Dublin 9, Ireland*

E-mail: zhussupa@tcd.ie

Methodology of in situ precursors decomposition

It should be noted that the magnitude of the reflection anisotropy spectroscopy (RAS) signal can vary significantly between different runs of the experiment as the ratio of the area containing the Au-dimers and the plain substrate can change due to limited degree of freedom in in alignment. In addition the self organized nature of the nanoparticles deposition process leads to variation of nanoparticles density within the functionalized area. The shape and amplitude of the RAS spectra will also change as function of temperature and total coverage of material already deposited on the sample. To allow for a reliable determination of the growth rate a normalization has to be employed.

The *in situ* measurements on the precursor decomposition was performed using low molarity solutions to maintain sufficiently low growth rates in order to keep the film accumulation during the *in situ* test as low as possible.

For every temperature and precursor combination aimed for investigation a spectra at the desired growth temperature is taken. Based on this spectrum an energy range with a local linear change of the RAS signal with energy with slope S_{spec} is identified. Increasing the refractive index between the nanoparticles in a subsequent growth run will, in a first order approximation, shift the plasmonic resonance towards the infrared. When measuring a transient at an energy range with a linear slope in the spectrum this results in a linear relationship of the RAS signal at this energy with increase in refractive index n. This rise in a RAS signal during the transient measurement represents an increase in material deposited over and between the gold dimers and we determine the slope of this change S_{trans}. By normalizing the latter with the spectral slope we get a re-normalized value $S_n = S_{\text{trans}}/S_{\text{spec}}$ which is comparable between subsequent steps, even if amplitude and shape of the plasmonic resonance changes with deposition steps and temperatures. As long as the total amount of material is significantly smaller than the height and separation of the Au-nanoparticles (h=40 nm and s=100 nm), S_n is proportional to a product of the films growth rate and refractive index.

As bulk samples are grown at typically higher molarities to increase growth rates, the proportionality of the growth rate for both precursors at the standard growth conditions for Cu_xCrO_2 was tested. The linearity of the growth was confirmed

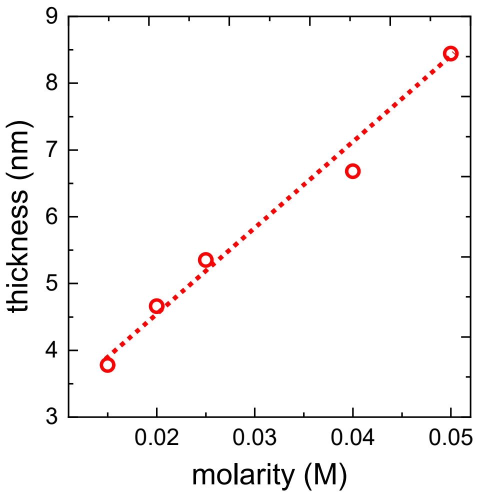

Figure S1: Growth rate vs. molarity for Cu_2O films grown from solutions with different $Cu(acac)_2$ molarity at 310°C.

by separate growth of films from $Cu(acac)_2$ and $Cr(acac)_3$ precursors at different molarities. Figure S1 shows that, as expected the growth rate determined by measuring total film thickness after a longer run are proportional to the solution molarity.

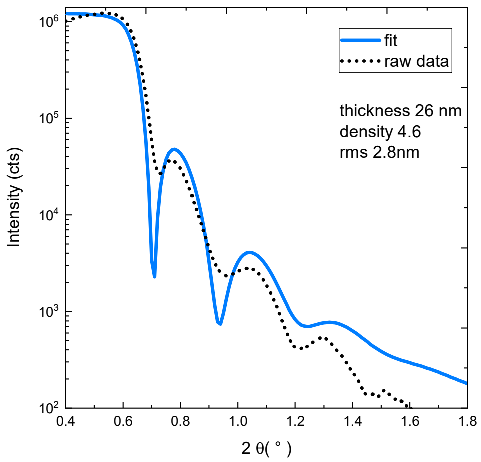

Figure S2: Representative XRR scan for one of the Cu_xCrO_2 films.

Samples characterisation

X-ray reflection method of thickness determination requires a low surface roughness, its use indicates the high quality of the films deposited via medical grade nebulizers. Figure S2 displays a representative XRR scan of the Cu_xCrO_2 films.

Performed Hall measurements demonstrate that it is not possible to extract reliable Hall signal value. This information however helps to set the limit for upper value for Cu_xCrO_2 mobility to be $\approx 0.1V^{-1}S^{-1}$.[1] It has been previously determined that small polaron hopping (SPH) is the dominating transport mechanism in the material. Therefore, to estimate hole mobility we measured the Seebeck coefficient. It was found to be in the 80–110 μ VK^{-1} range for the Cu_xCrO_2 films.

The Seebeck coefficient S can be used to estimate the mobility of thin films within SPH model[2] as it is related to the fraction of occupied carrier cites c:

$$S = \pm\frac{K_B}{e}ln[\frac{2(1-c)}{c}], \quad (1)$$

rearranging this expression terms of c we get

$$c = exp[\frac{2}{Q_R+2}], \quad (2)$$

where Q_R is reduced Seebeck coefficient, Se/K_B. The calculated c can be used to estimate the carrier concentration p of the films via p=Nc, where N is the number of available carrier sites.[3]

Mobility is then calculated to be $1\times10^{-3}cm^2V^{-1}s^{-1}$ for a sample with conductivity of 1 Scm^{-1} and $8\times10^{-3}cm^2V^{-1}s^{-1}$ for a sample with conductivity of 12 Scm^{-1}.

Conductivity was determined from

$$\sigma = \frac{1}{R_{sh}\times d}, \quad (3)$$

where R_{sh} is the measured sheet resistance determined from Hall measurement in Van der Pauw geometry, d is film thickness measured by X-ray reflection.

Carrier activation energy is measured in an experimental setup that consists of a Keithley 2400 source meter with electrodes being spring loaded gold probes. The samples were heated by a Watlow ceramic heater and the thin-film resistivity measured in situ while the sample temperature varied controlled by a PID controller. Resistive heating allows the low temperature annealing and the

activation energy E_a measurements via the use of the following relation

$$R_{sh} = R_0 \times exp - (\frac{E_a}{kT}) \tag{4}$$

The sheet resistance measurement is carried through four linearly spaced probes that are brought into contact with the film surface. The current is applied through two outer contacts and generated potential can be measured via the two inner probes. The measured sheet resistance is described by the following expression:

$$R_{sh} = \frac{\pi}{ln(2)} \frac{V}{I} \tag{5}$$

The XPS cation ratio used in the Figure S3 in the main paper was determined from high resolution scans of the Cu 2p and Cr 2p core levels with Cu $2p^{3/2}$ and Cr $2p^{3/2}$ chosen for are analysis in Casa XPS. The resulted ratio of the elements presented as Cu content/(Cu+Cr) in atomic %.

References

(1) Nagaraja, A. R.; Perry, N. H.; Mason, T. O.; Tang, Y.; Grayson, M.; Paudel, T. R.; Lany, S.; Zunger, A. Band or Polaron: The Hole Conduction Mechanism in the p-Type Spinel Rh2ZnO4. *Journal of the American Ceramic Society* **2011**, *95*, 269–274.

(2) Mansourian-Hadavi, N.; Wansom, S.; Perry, N. H.; Nagaraja, A. R.; Mason, T. O.; hui Ye, L.; Freeman, A. J. Transport and band structure studies of crystallineZnRh2O4. *Physical Review B* **2010**, *81*.

(3) Farrell, L.; Norton, E.; Smith, C. M.; Caffrey, D.; Shvets, I. V.; Fleischer, K. Synthesis of nanocrystalline Cu deficient CuCrO2-a high figure of merit p-type transparent semiconductor. *Journal of Materials Chemistry C* **2015**, *4*, 126–134.

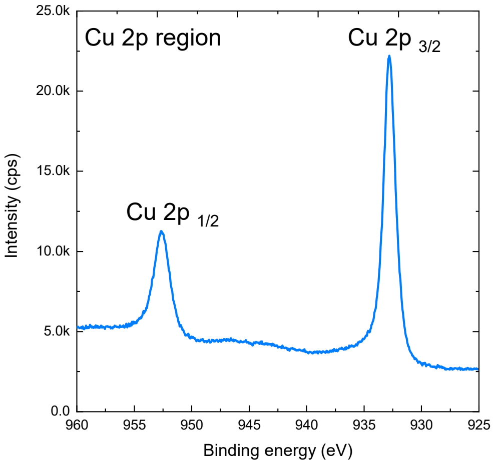

(a)

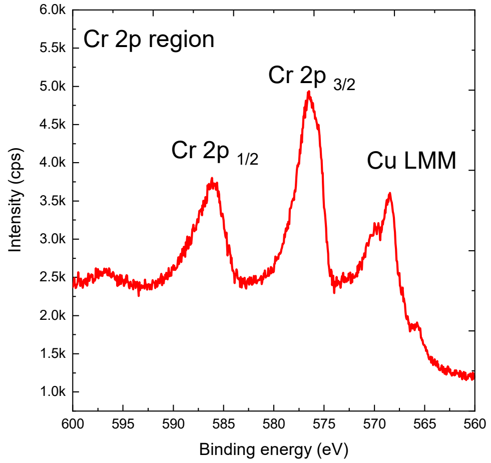

(b)

Figure S3: Representative XPS scans of the Cu 2p region (a) and Cr 2p region (b) employed in the Cu/Cr ratio determination for spray grown Cu_xCrO_2 films.
